# Using the Autism-Spectrum Quotient to Measure Autistic Traits in Anorexia Nervosa: A Systematic Review and Meta-Analysis

**DOI:** 10.1007/s10803-015-2641-0

**Published:** 2015-11-05

**Authors:** Heather Westwood, Ivan Eisler, William Mandy, Jenni Leppanen, Janet Treasure, Kate Tchanturia

**Affiliations:** Psychological Medicine, IoPPN, King’s College London (KCL), PO59, London, SE5 8AF UK; Child and Adolescent Eating Disorders Service, Maudsley Hospital, London, SE5 8AZ UK; Department of Psychology, IoPPN, King’s College London (KCL), London, UK; Research Department of Clinical, Educational and Health Psychology, University College London (UCL), London, UK; Ilia State University, Tbilisi, Georgia

**Keywords:** Autism, Systematic review, Meta-analysis, Anorexia Nervosa, Autism-Spectrum Quotient, Female

## Abstract

Interest in the link between Autism Spectrum Disorder (ASD) and Anorexia Nervosa (AN) has led to estimates of the prevalence of autistic traits in AN. This systematic review and meta-analysis assessed the use of the Autism-Spectrum Quotient (AQ) or abbreviated version (AQ-10) to examine whether patients with AN have elevated levels of autistic traits. Seven studies were identified and subsequent meta-analysis indicated that those with AN appear to have significant difficulties of a manner characteristic of ASD, relative to controls. Whilst this analysis supports previous indications of higher prevalence of ASD in AN, the aetiology of these traits remains unclear. Studies using more robust clinical measures of ASD within AN are needed to confirm what self-report measures appear to show.

## Introduction

Research into the possible link between Autism Spectrum Disorders (ASD) and Anorexia Nervosa (AN) has grown considerably, with studies showing potential links both in specific characteristics of the two disorders (Oldershaw et al. 2011) and in prevalence rates of co-morbidity or elevated levels of ASD traits within AN populations (for a systematic review see Huke et al. 2013).

AN is a severe eating disorder characterised by body weight significantly lower than the normal range relative to height, fear of gaining weight and undue influence of weight and shape on self-evaluation (APA 2013). It predominantly affects females, with an estimated gender ratio of 10:1 females to males, though epidemiological studies report higher variation of 3:1–12:1 (Raevuori et al. 2014). In contrast, ASD is a pervasive developmental disorder that tends to affect more males than females, with a gender ratio of 3.3:1 males to females being reported in a UK national prevalence study (Mills and Kenyon 2013). Unlike AN, which tends to have its onset in adolescence to early adulthood (Micali et al. 2013), for ASD to be diagnosed, symptoms must be present during the early developmental period. These include persistent difficulties with social communication and interaction and restricted, repetitive behaviours and interests (APA 2013).

Despite these two disorders seeming different, several traits associated with ASD have also been found in AN populations including: difficulties with set-shifting (Tchanturia et al. 2012), the capacity to shift a course of thought or action according to situational demands (Lezak 1995); weak central coherence, the lack of an effect of context or difficulty in taking a global approach (Frith 1989; Lang et al. 2014); difficulties with Theory of Mind (ToM), the ability to infer the mental states of others (Baron-Cohen et al. 1985; Tchanturia et al. 2004); and difficulties with emotional processing (Davies et al. 2011; Russell et al. 2009).

Although there is a male bias in the diagnosis of ASD, recent evidence suggests that it may affect more females than previously thought, with diagnostic criteria and research practices leading to an over-stated gender gap (Goldman 2013; Lai et al. 2015). ASD may present differently in females than in males so that females are often under-diagnosed or mislabelled as having other impairments (Mandy and Tchanturia 2015). For example, females with ASD have been found to have better social skills than their male counterparts (Head et al. 2014) and females with ASD out-perform males on tests of executive functioning (Bolte et al. 2011). Within ASD, typical sex differences between males and females on empathising and systemising are attenuated, with both sexes shifting towards the extreme male brain (Baron-Cohen et al. 2014). However, this study found that significant sex differences within ASD still exist, in the same direction as Healthy Controls (HCs), highlighting the need to consider sex-differences in the diagnosis of ASD.

Females may also display less stereotyped behaviours or restricted interests (Mandy et al. 2012) or their interests may be more subtle or in line with gender stereotypes, making them more difficult to detect (Hiller et al. 2015). While AN tends to develop during adolescence, it may be that the presence of undetected ASD traits in early life interact with socio-cultural pressures to leave females in particular, susceptible to the development of eating disorders. Difficulties with set-shifting and central coherence are consistently reported in adults with AN (for a review, see Roberts et al. 2007) but the evidence in children and adolescents is more mixed (Lang et al. 2014a, b). However, this may be due to several studies using different experimental paradigms to measure central coherence and set-shifting and under-powered studies, which make the data difficult to interpret or compare. It is therefore still unclear whether children with AN, who are exposed to the effects of starvation for less time, have the same cognitive inefficiencies as adults. Findings on ToM are also mixed, with research not supporting a specific link between ToM and AN (Tchanturia et al. 2004). Research on emotional ToM in AN suggests that problems in this area resolve with weight gain and recovery, supporting the notion that this difficulty is at least exacerbated by the ill state of the disorder (Oldershaw et al. 2010).

The marked overlap in specific characteristics present in both ASD and AN has led to attempts to examine the prevalence of co-morbid ASD or elevated levels of autistic traits within AN. As ASD is considered a dimensional disorder (Bolton et al. 1994; Wiggins et al. 2012) a distinction can be made between ASD as a fully diagnosed clinical disorder and the presence of sub-clinical traits, intermediate between typical functioning and ASD, i.e. elevated levels of ASD traits found in the general population. Studies with AN populations have examined the presence of both co-morbid ASD and elevated levels of autistic traits using several instruments. Studies assessing the prevalence of ASD have tended to use diagnostic criteria (Gillberg et al. 1995; Nilsson et al. 1998, 1999; Rastam 1992; Rastam et al. 2003) and The Asperger Syndrome Diagnostic Interview (Anckarsater et al. 2012; Nilsson et al. 1999; Rastam et al. 2003). A meta-analysis of these prevalence studies found an average ASD prevalence rate of 22.9 % (Huke et al. 2013) in eating disorder populations. The majority of these studies, however, involved the same Swedish community sample and the variety of diagnostic tools used makes it difficult to compare the outcomes. The review only contained studies with adults with AN whereas a prevalence study examining ASD in adolescents with early onset-AN found that a diagnosis of ASD was no more common than in healthy controls (Pooni et al. 2012), although the AN sample did have elevated levels of ASD traits.

A widely used measure of autistic traits is the Autism-Spectrum Quotient (AQ; Baron-Cohen et al. 2001) which was developed to provide a brief, self-report measure of autistic traits for use with adults, rather than for use as a diagnostic tool. When the AQ was initially developed, individuals with high functioning autism (HFA) were found to have a mean score of 35.8 (SD = 6.5) which was significantly higher than the control group who had a mean score of 16.4 (SD = 6.3). Among the controls, but not in the HFA group, men were found to score significantly higher than women. For the original validation study, a cut-off score of 32 was chosen as 80 % of those with HFA scored above this level, while only 2 % of controls did. Test–retest and inter-rater reliability of the AQ were good, and a cut-off score of 32 has acceptably high sensitivity (0.77) and specificity (0.74) (Austin 2005; Woodbury-Smith et al. 2005).

The AQ is a 50-item questionnaire consisting of five domains: social skills; attention switching; attention to detail; communication and imagination. The AQ has reasonable face validity as only 2 % of control participants scored above the clinical cut-off during the original validation study (Baron-Cohen et al. 2001). Items comprising each of the five domains showed moderate to high alpha coefficients, indicating reasonable construct validity. Additional analysis also found the AQ to have moderate accuracy in diagnosis of Asperger’s Syndrome in clinical settings, i.e. correctly identifying a ‘true positive’ case as scoring higher than a ‘true negative’ case (Woodbury-Smith et al. 2005).

In addition to the AQ being used as a screening tool for ASD in the general population, it has been used to a examine ASD traits within clinical groups. For example, in schizophrenia (Mealey et al. 2014; Spek and Wouters 2010; Wouters and Spek 2011) and obsessive compulsive disorder (Cath et al. 2008; Mito et al. 2014). The criterion validity of the AQ has been found to be good, with patients with HFA scoring considerably higher than those with social anxiety disorder or obsessive compulsive disorder (Hoekstra et al. 2008). This demonstrates that the AQ can differentiate between individuals with ASD and other disorders.

To examine whether individuals with AN have elevated levels of ASD traits, relative to the general population, several studies have utilised the AQ or the abbreviated ten-item version, the short Autism Spectrum Quotient (AQ-10; Allison et al. 2012). The AQ-10 has similar sensitivity and specificity to the full version and performed well as a screen for ASD (Booth et al. 2013), with a clinical cut-off of 6 being indicative of ASD. If validated, self-report measures such as the AQ or AQ-10 have the benefit of being cost-effective and time efficient and thus could provide a useful addition to more robust but time-consuming clinical assessments.

The presence of ASD in AN represents a significant treatment challenge (Nielsen et al. 2015) and thus identifying ASD traits within this population is important. However, disentangling the relationship between AN and ASD has proved problematic. The presence of ASD traits in AN appear to differ across different stages of the illness, suggesting that these traits are epiphenomena, arising as a result of the ED and only superficially resembling ASD (Mandy and Tchanturia, Additionally, ASD traits in females are difficult to detect and the presence of a severe eating disorder further complicates the identification and diagnosis of ASD in this group. The presence of eating disorders in individuals with established ASD diagnoses has received less attention than ASD in AN populations. Eating disturbances, including selective eating, are known to be overrepresented in ASD (Rastam 2008) and may serve as a risk factor for the development of a clinical eating disorder, such as AN. Karlsson et al. (2013) note that eating disturbances in ASD are clinically acknowledged but under-researched. Thus, disentangling the relationship between the two disorders is difficult and it is important to establish the most robust method of identifying ASD traits within AN populations.

The aims of this systematic review and meta-analysis were to: (1) examine whether patients with AN have elevated levels of self-reported autistic traits relative to HCs and (2) compare the use of the AQ and AQ-10 in studies with AN populations (3) Examine whether the AQ or AQ-10 are robust measures of ASD traits in AN populations, given the presence of other co-morbidities, the impact of the eating disorder on these traits and the stability of ASD traits during the course of AN.

## Methods

The meta-analysis was conducted according to the ‘PRISMA’ (preferred reporting items for systematic reviews and meta-analysis) statement (Moher et al. 2009).

### Eligibility Criteria

Studies using the AQ or AQ-10 with both a clinical AN and HC group were included in the review. Other eligibility criteria included being published in a peer-reviewed journal and being available in English.

### Information Sources and Search

The electronic databases Scopus, Psychinfo, Medline and Web of Science were searched up to and including April 2015. Search terms included AQ, Autism, ASD, eating disorder and Anorexia Nervosa. Limits included English language, articles and peer-reviewed. Reference lists of eligible papers were also screened for other relevant studies and additionally, Lang et al. (2015) was included (data were available from the principal author of the study, KT). Missing data on AQ subscales were also obtained directly from the authors of one study (Huke et al. 2014).

### Selection

The first and principal authors (HW, KT) identified potential titles from all databases and screened the abstracts for relevance. Full-texts were then retrieved and read to determine eligibility. Texts deemed eligible were then further screened by KT in discussion with HW and any papers that did not meet inclusion criteria were excluded.

### Data Collection and Items

The data items collected from each eligible study were: number of participants, age of participants, BMI, illness duration (for the AN group), percentage of female participants, how HCs were matched to clinical samples, use of medication, co-morbidities, IQ and the version of the AQ used i.e. full or AQ-10. In addition to demographic and experimental paradigm data, the mean and standard deviations of the AQ or AQ-10 score were used within the meta-analysis. In studies using the full AQ, means and standard deviations of the AQ sub-scale scores for AN and HC groups were also extracted so that these could be statistically compared across studies.

### Risk of Bias in Individual Studies

The risk of bias in individual studies was assessed by considering how methodology would impact on effect size in each study, for example by attending to how healthy controls were matched to clinical samples, how the AQ or AQ-10 were administered and the source of participant recruitment.

### Summary Measure

The principal summary measure used for analysis from all studies was the difference in means and standard deviations of AQ or AQ-10 scores between the AN sample and HCs.

### Synthesis of Data

Synthesis of results for meta-analysis meant that only studies reporting mean and standard deviation AQ or AQ-10 scores were included in the analysis. The meta-analysis was performed by pooling the standardised effect sizes using a random effects model. Random effects models assume that as well as within-group variability caused by variability of scores, mean effect size is also caused by differences between studies. The model includes the between study heterogeneity, resulting in estimates with wider confidence intervals than fixed-effects models. Additional meta-analyses, again using random-effects model were conducted to compare AN patients and HCs on sub-scale scores of the AQ (social skills, attention switching, attention to detail, communication and imagination).

### Statistical Analysis

Analysis was carried out in STATA 11 (StataCorp, College Station, TX) with the following user contributed commands *metan* (Bradburn et al. 1998), *metabias, metatrim* (Steichen 1998), and *metareg* (Sharp 1998). For all meta-analyses Cohen’s *d* was used to estimate effect size and is reported for all studies together with 95 % confidence intervals. The effect sizes were interpreted according to Cohen’s (1988) definitions of small (≥ 0.20 ≤ 0.50), medium (≥ 0.50 ≤ 0.80), large (≥ 0.80 ≤ 1.30) and very large (≤1.30). Positive effect size indicates that the AN group had higher scores on the AQ or AQ-10 than HC. A *p* value of <0.05 indicates significant difference between groups. For sub-scale analysis, Review Manager 5.3 (Cochrane Collaboration 2014) was used.

### Risk of Bias Across Studies

Publication bias was assessed by evaluating funnel plots of each study’s mean differences. Symmetry of the funnel plot was assessed visually and by Egger’s test (Egger et al. 1997) to see if sample size was related to effect size.

### Additional Analysis

Between studies heterogeneity was measured calculating *I*^2^ (Higgins et al. 2003) based on Cochran’s Q test: measure of heterogeneity, I^2^ = 100 % (Q-df)IQ. *I*^2^ ranges between 0 %, indicating no heterogeneity and 100 %, indicating high heterogeneity. Where heterogeneity was found a meta-regression was performed with age as a moderator. It was not possible to conduct a meta-regression with either BMI or gender as moderator variables due to missing data on BMI and all but one study included only female participants. Meta-regression of sub-scale scores was also not possible due to only four studies reporting this data.

## Results

### Study Selection

**Fig. 1:**
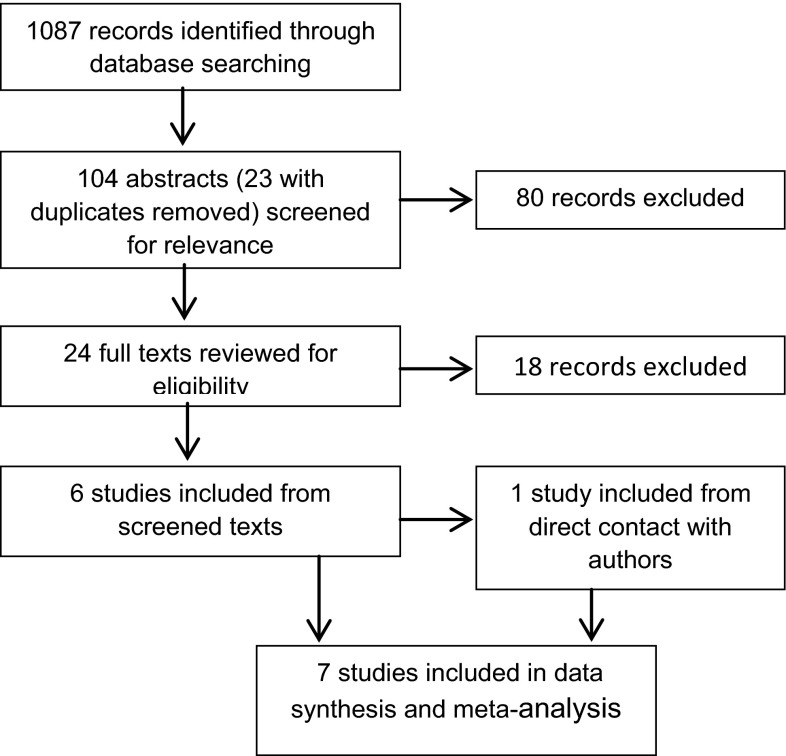
Systematic review search process in accordance with the PRISMA statement

A total of seven studies were identified and included in the review, consisting of a pooled total of 328 AN patients and 1890 HCs. Of the AN sample, 90 were children or adolescents. Two of these studies (Baron-Cohen et al. 2013; Lang et al. 2015) had multiple data sets for adult and child/adolescent samples resulting in nine data sets for the meta-analysis. Although the adult and child data are pooled within the Lang et al. (2015) paper, at our request the authors provided data separately for these groups. The selection process for studies is shown in Fig. 1. The main reasons for exclusion of studies were: the AQ or AQ-10 was not used to measure ASD traits, the clinical sample did not have a primary diagnosis of AN, the papers did not contain empirical data or the papers were not available in English.

### Study Characteristics

Studies included in the systematic review and meta-analysis are displayed in Table 1. Five of the seven included studies (Baron-Cohen et al. 2013; Calderoni et al. 2015; Courty et al. 2013; Hambrook et al. 2008; Huke et al. 2014) used the full AQ, while the remaining two studies (Lang et al. 2015; Tchanturia et al. 2013) used the AQ-10. Of the five studies using the AQ, four collected data on the sub-scale differences between the AN and HC samples (Calderoni et al. 2015; Courty et al. 2013; Hambrook et al. 2008; Huke et al. 2014).

**Table 1:** Participant demographic information for included studies

Author/date	Group	N	Age M (SD)	BMI M (SD)	Illness duration M (SD)	AQ version	AQ score M (SD)	Female (%)	Groups matched by
*Adult studies*									
Hambrook et al. (2008)	AN	22	26.73 (4.77)	15.27 (1.22)	9.5 (5.0)	Full	23.2 (7.3)	100	Gender
	HC	45	32.51 (9.93)	23.36 (3.76)			15.3 (5.5)	100	
Courty et al. (2013)	AN	15	23.9 (4.7)	16.4 (1.7)	4.0 (3.5)	Full	20.3 (5.9)	93.3	Age, gender, education
	HC	15	24.0 (4.9)	21.0 (1.8)			14.8 (4.9)	93.3	
Baron-Cohen et al. (2013)	AN	42	17.85 (0.38)*			Full	21.0 (6.8)	100	Gender
	HC	1038	18.56 (3.99)*				15.5 (5.6)	100	
Tchanturia et al. (2013)	AN	66	26.35 (8.08)	14.90 (2.13)	10.59 (6.66)	AQ-10	4.53 (2.56)	100	Gender
	HC	66	25.68 (9.74)	21.78 (2.46)			1.85 (1.68)	100	
Huke et al. (2013)	AN	32	28.7 (9.65)	14.71 (1.77)	11.03 (9.33)	Full	20.03 (9.65)	100	Age, gender
	HC	32	24.9	22.5 (3.22)			14.78 (6.23)	100	
Lang et al. (2015)	AN	61	26.72 (7.61)	15.44 (1.68)	8.74 (7.84)	AQ-10	4.05 (2.22)	100	Gender
	HC	69	27.30 (9.29)	22.34 (2.42)			1.52 (1.24)	100	
*Child/adolescent studies*									
Baron-Cohen et al. (2013)	AN	24				Full	21.0 (6.8)	100	Gender
	HC	412					15.5 (5.6)	100	
Lang et al. (2015)	AN	41	15.07 (1.81)	80.69 % (6.57)**	1.70 (1.18)	AQ-10	3.88 (2.12)	100	Gender
	HC	43	15.11 (1.94)	100.92 % (8.37)**			2.67 (1.28)	100	
Calderoni et al. (2015)	AN	25	14.34 (1.86)	14.63 (1.90)		Full	21.04 (6.52)	100	Age, gender, education
	HC	170	15.01 (2.23)	19.75 (2.61)			17.46 (5.50)	100	

*N* number of participants, *BMI* body mass index (kg/m^2^)

* Overall mean age for whole sample including children and adolescents, adult mean age not available

** % weight for height

In all studies, groups were matched by gender and in all but one study (Courty et al. 2013), all participants were female. In three studies, the AN sample was also matched to HCs by age (Calderoni et al. 2015; Courty et al. 2013; Huke et al. 2014) and in two studies, by level of education (Calderoni et al. 2015; Courty et al. 2013). In four of the studies (Baron-Cohen et al. 2013; Hambrook et al. 2008; Lang et al. 2015; Tchanturia et al. 2013) AN diagnosis was made in accordance with DSM-IV criteria (APA 1994) while two studies used DSM-IV-TR (APA 2000) criteria (Calderoni et al. 2015; Courty et al. 2013). One study did not state which diagnostic criteria were used, but diagnosis was checked using the Eating Disorder Examination (12th Ed, Fairburn and Cooper 1993). Calderoni et al. (2015) included only patients with Anorexia Nervosa restricting subtype (AN-R) whilst the other studies either did not specify or included a mixture of both binge/purge and restricting type AN.

The studies included in the overall AQ score meta-analysis differed greatly in terms of their sample size. For example, Baron-Cohen et al. (2013) had an AN sample of 66 but a HC sample of 1609, so power calculations would only account for the smaller, AN sample. The smallest sample size was 15 in Courty et al. (2013)’s study and Lang et al. (2015)’s study had the largest, most equal sample size of 96 and 97 AN and HCs respectively. Although BMI was reported by all but one study (Baron-Cohen et al. 2013), two studies did not report AN illness duration (Baron-Cohen et al. 2013; Calderoni et al. 2015) so this could not be used as a moderator variable when examining heterogeneity across studies.

The use of medication by study participants was only reported in one study (Courty et al. 2013) who reported that 65.4 % of AN patients were taking psychotropic medication. Attempt was made by all studies to control for psychiatric illness within both the AN and HC groups. Baron-Cohen et al. (2013) excluded one participant with AN whom also had ASD and used a self-report questionnaire with HCs to assess for psychiatric illness. Courty et al. (2013) ruled out ASD in the AN and HC groups and found that the AN group had higher levels of depression than the HCs. Calderoni et al. (2015) excluded anyone who displayed psychotic symptoms, had substance abuse and those with an IQ of below 80. In the AN group, 80.8 % met criteria for an anxiety or mood disorder and two patients had a co-morbid personality disorder. Huke et al. (2014) excluded participants with psychosis, drug and alcohol misuse or those with a high risk of suicide. HCs were also excluded if they had a current or previous diagnosis of a mental disorder, as was the case in Tchanturia et al. (2013). Hambrook et al. (2008) screened for psychotic disorder in the HC group. IQ was only assessed in one study (Lang et al. 2015) and years of education were recorded in two studies (Huke et al. 2013; Tchanturia et al. 2013), with no significant differences being found between the AN and HC groups.

There were also differences between the studies in how the AQ or AQ-10 was administered. For example, due to the self-report version of the AQ only being validated for children over the age of 16, the parent version of the AQ was used for younger participants in Baron-Cohen et al. (2013)’s study. Baron-Cohen also sent the questionnaires by post, as did Courty et al. (2013). In all other studies, participants completed the questionnaires during a testing session. Whilst five of studies took place in the United Kingdom (Baron-Cohen et al. 2013; Hambrook et al. 2008; Huke et al. 2014; Lang et al. 2015; Tchanturia et al. 2013), one study was conducted in Italy (Calderoni et al. 2015) and one in France (Courty et al. 2013). AN samples were also recruited from a variety of services: three studies recruited from a mixture of outpatient, day patient and inpatient services within the same, national specialist eating disorder service (Hambrook et al. 2008; Lang et al. 2015; Tchanturia et al. 2013), three only recruited from inpatient services (Calderoni et al. 2015; Courty et al. 2013; Huke et al. 2014) and one states that patients were recruited from a specialist service but does not state whether this was an inpatient setting (Baron-Cohen et al. 2013).

### Risk of Bias

**Fig. 2:**
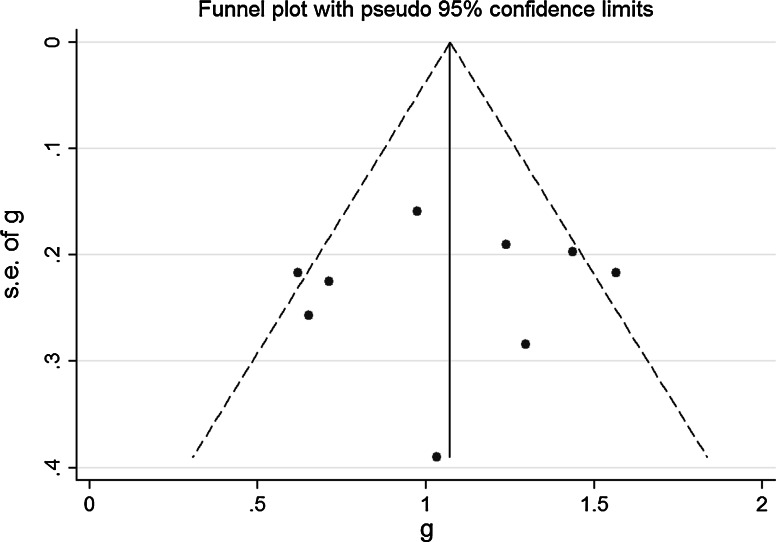
Funnel plot of studies included in the meta-analysis to assess for publication bias. *Each dot* represents a study included in the meta-analysis, with the Y axis representing the size of each study and the X axis, each study’s result

There was good symmetry within the funnel plot, shown in Fig. 2, indicating no relationship between effect and study size. Additionally, Egger’s test was conducted to statistically assess for publication bias, indicating no evidence of bias (*p* = 0.829).

### Synthesis of Results

**Fig. 3:**
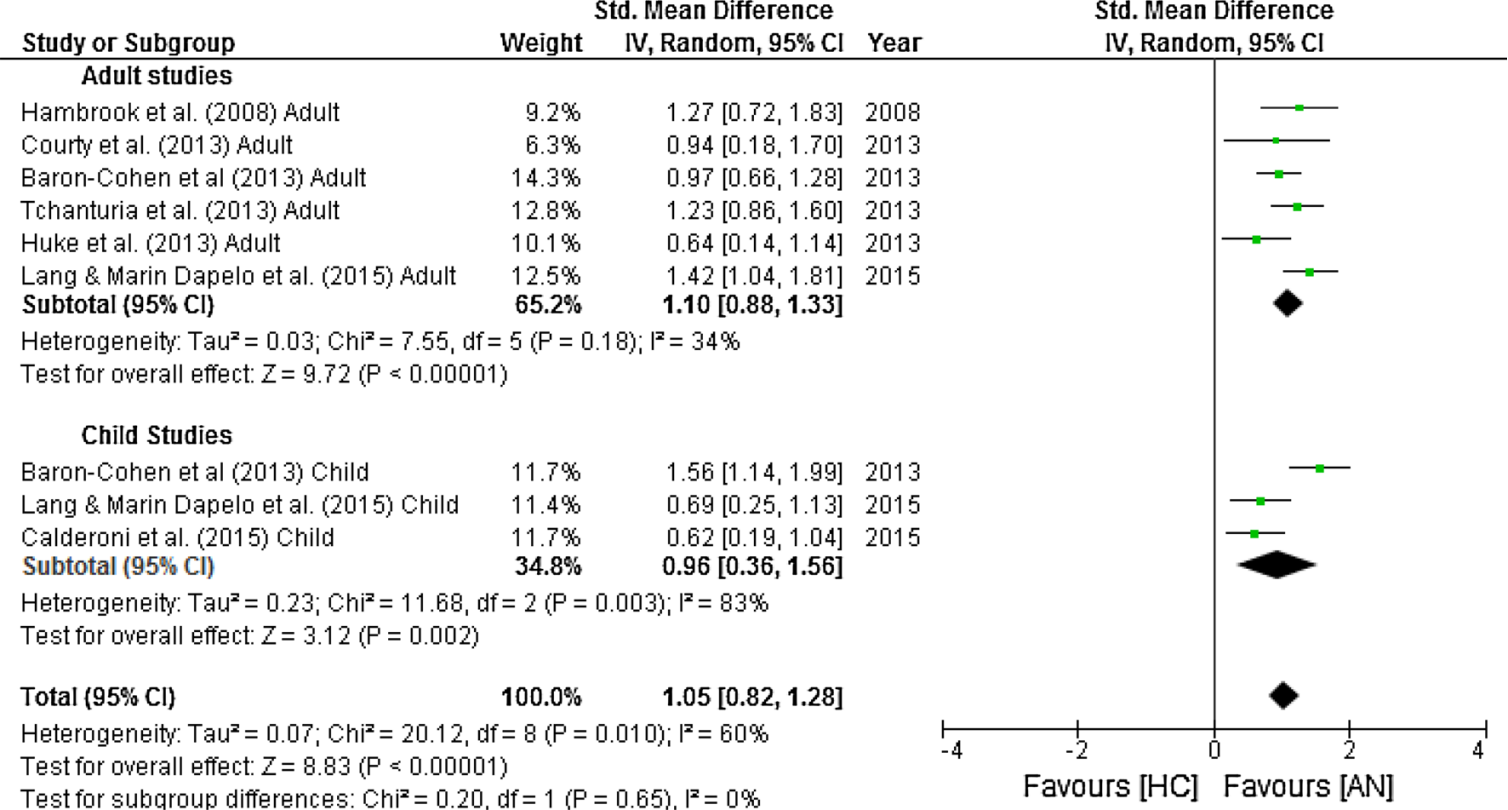
Forest plot for mean total Autism-Spectrum Quotient (AQ) and short Autism-Spectrum Quotient (AQ-10), with standardised mean effect sizes for difference between Anorexia Nervosa (AN) and Healthy Controls (HC). The Y axis shows each included study and the X axis represents the magnitude of difference between the AN and HC groups. The overall effect sizes for adult and child studies and the mean summary measure are depicted by the *black diamonds*

The forest plot of all studies included in the overall AQ or AQ-10 score meta-analysis is displayed in Fig. 3. The random-effects analysis, with a total sample size of 2218 (AN = 328, HC = 1890) revealed a significant difference between AN and HC groups on the AQ or AQ-10 mean scores, d = 1.065 [95 % CI: 0.83, 1.23], z = 8.90, *p* = <0.001.

**Fig. 4:**
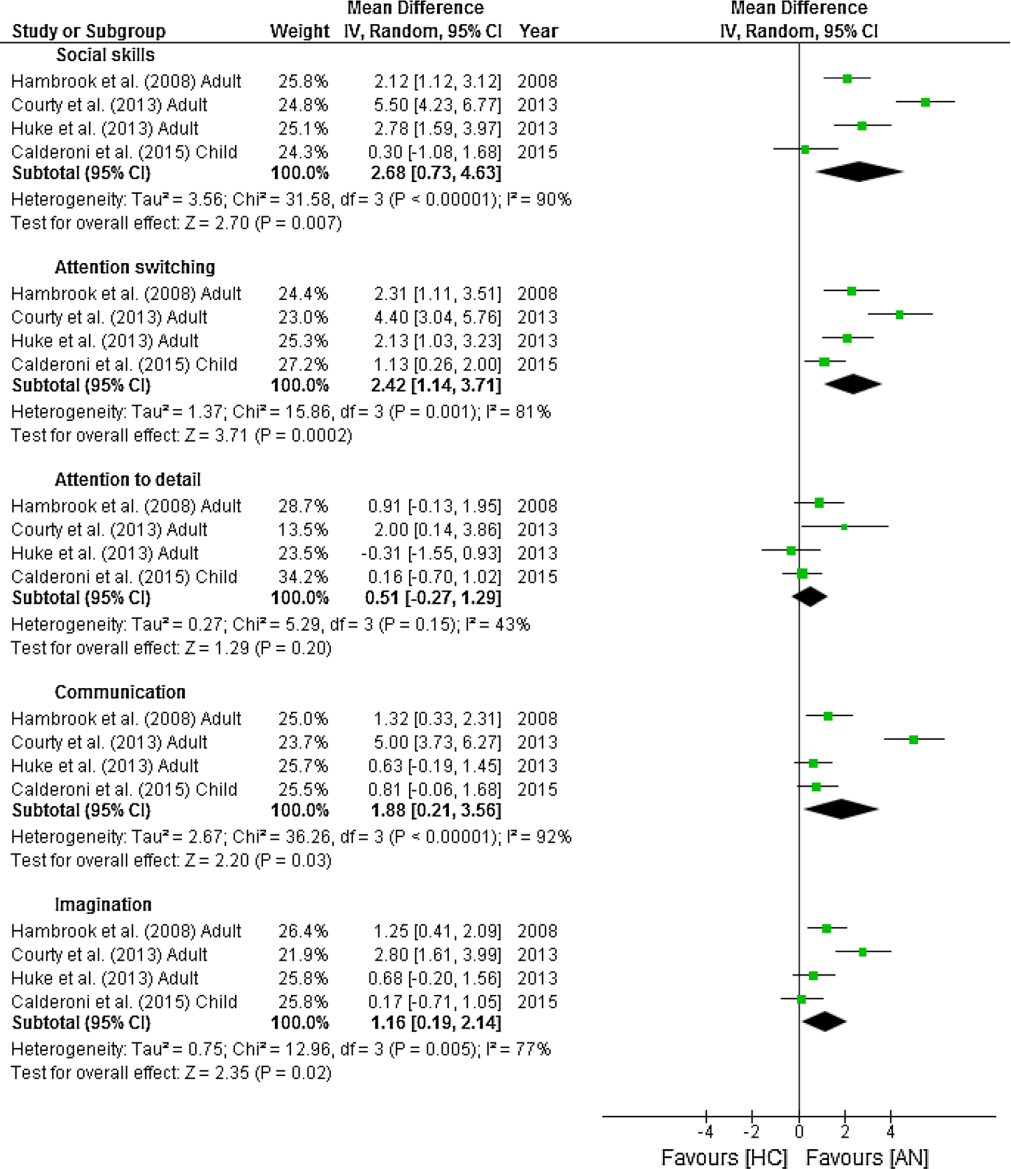
Mean and standard deviations sub-scale scores and forest plots for Autism-Spectrum Quotient with effect sizes for difference between Anorexia Nervosa (AN) and Healthy Controls (HC). The Y axis shows each individual study and the X axis represents the magnitude of difference between the AN and HC groups on the AQ sub-scales. The overall effect sizes for each sub-scale score are depicted by the *black diamonds*

Forest plots for the AQ sub-scale analysis are displayed in Fig. 4. The random-effects analysis, with a total sample size of 358 (94 AN, 262 HCs) revealed significant differences between AN and HCs on four of the five subscales; social skills, attention switching, communication and imagination. Social skills: MD = 2.68 [95 % CI: 0.73, 4.63], z = 2.7, *p* = 0.007. Attention switching: MD = 2.42 [95 % CI: 1.14, 3.71], z = 3.71, *p* = 0.0002. Attention to detail: MD = 0.51 [95 % CI: −0.27, 1.29], z = 1.29, *p* = 0.20. Communication: MD = 1.88 [95 % CI: 0.21, 3.56], z = 2.20, *p* = 0.03. Imagination: MD = 1.16 [95 % CI: 0.19, 2.14], z = 2.35, *p* = 0.02.

### Additional Analysis

There was evidence of considerable heterogeneity between studies included in the total score meta-analysis (*I*^2^ = 59.8 %). A meta-regression was conducted to further investigate the variance, with age as a moderator. The meta-regression showed no significant effect of age on the unexplained between-study variation. Due to incomplete data, meta-regression using other moderator variable was not possible but the potential reasons for this heterogeneity are explored within the discussion. Moderate to high heterogeneity was also found between studies included in AQ-subscale meta-analysis (*I*^2^ = 43 % to 92 %). However, due to the small number of studies, this could not be examined further statistically.

## Discussion

The aim of this review was to provide a synthesis of the existing literature on the presence of autistic traits in AN, as measured by the self-report AQ or AQ-10. It also aimed to establish whether factors such as age or illness severity predict AQ scores and thus whether the AQ is a robust measure of ASD traits in AN populations. A number of methodological differences across studies were highlighted, including the differing use of either the full AQ or shortened AQ-10, differences in sample size and the way in which confounding factors such as BMI, illness duration, medication and co-morbidities were controlled for within the studies. Data on BMI, gender and illness duration were not sufficient to allow for meta-regression to explore whether these factors predict AQ scores. However, generally, the studies all reported enough information to allow for synthesis and analysis of the data.

The meta-analysis indicates a significant difference between AN patients and HCs on the scores on both the AQ and AQ-10, with AN patients scoring significantly higher, suggesting significant difficulties with social skills, communication and flexibility that present in a manner characteristic of autistic traits. This finding is in line with previous prevalence research in this area, for example the systematic review by Huke et al. (2013) which reported a mean ASD prevalence rate of 22.9 % in AN, higher than that prevalence of ASD within the general population which is estimated to be 1.1 % in the UK (Mills and Kenyon 2013). However, the findings of this meta-analysis should be interpreted with some caution.

All patients included in the studies were within the acute, ill phase of AN with BMIs considerably lower than the normal range. The highest mean BMI for the clinical group was 16.4, recorded by Courty et al. (2013); this is considered to be moderate in terms of severity (APA 2013). The lowest mean BMI was reported by Calderoni et al. (2015) at 14.63, which is considered to be extreme AN (APA). Thus, although in both of these studies patients were recruited from inpatient settings, the severity of the AN may have been different and thus severity of AN may have account for the heterogeneous samples included in this review. Countries differ on the treatments offered to patients at different severities. Even in the UK, where five of the studies were based, there is large variation across services in the treatment patients receive, even within the moderate to severe range of AN (Goddard et al. 2013).

Severity of the eating disorder is an important consideration due to the possibility of ASD traits being exacerbated by starvation or the acute state of the illness (Pellicano and Hiller 2013). With the mean duration of illness across the included studies also varying from 4 to 11 years, the possibility of illness chronicity affecting the results of the AQ cannot be ruled out. As the AQ focuses on current behavioural symptoms, rather than on ASD traits earlier on in life, it is possible that individuals with AN develop autistic-like traits as a result of their eating disorder. Lack of data meant that exploring the impact of BMI or illness duration on AQ scores was not possible within this review. While none of the included studies make claims about the aetiology of elevated autistic traits in AN, this is an important treatment consideration and warrants further investigation.

A study comparing the AQ in ASD and schizophrenia (Lugnegård et al. 2015) found that while individuals with either ASD or schizophrenia score high on the AQ, nine individuals with schizophrenia who also met childhood criteria for ASD did not show significantly higher scores on the AQ than the individuals without ASD. This suggests that the AQ may not be sensitive in differentiating between individuals with ASD and those with other psychiatric disorders. Additionally, it may suggest that AQ scores are not stable over time in psychiatric populations. Research on the stability of autistic traits during development (Whitehouse et al. 2011) found that while these traits appeared to be stable in males, with scores in childhood being correlated with those in adulthood, this was not the case in females. Again, this indicates that autistic traits may present and develop differently in females, making it difficult to interpret whether the elevated AQ scores seen in AN females are a true reflection of elevated autistic traits in this population.

Although age was used within the meta-regression in an attempt to explain heterogeneity between the studies, this was not found to be significant. Despite this, only three studies (Baron-Cohen et al. 2013; Calderoni et al. 2015; Lang et al. 2015) included participants below the age of 18 and when child/adolescent studies were analysed separately, the effect size was smaller. There is a lack of consensus about whether difficulties such as those present in ASD, including social communication and flexibility, are present prior to the onset of the AN or whether they are state dependent, resulting from starvation and an enduring illness and thus not truly autistic in origin (Pellicano and Hiller 2013). Therefore, although the results of this meta-analysis suggest elevated levels of autistic traits in AN, the aetiology of these apparent traits remains obscure. Smaller effect sizes in studies with children and adolescents with AN may indicate less difficulties associated with ASD. This would be concordant with other research in this area, which suggests that the similarities between AN and ASD may be less pronounced in children (Lang et al. 2014a, b; Pooni et al. 2012; Rhind et al. 2014). However, research in this area is not robust enough to confirm this.

Despite the self-report version of the AQ not being validated with children under the age of 16, only one of the child studies included in this review (Baron-Cohen et al. 2013) used the parental AQ version for younger participants. It is therefore possible that the smaller effect found in child/adolescent studies are due to methodological differences between studies. Given the limitations of current data, interpretation of any differences in findings between adults and young people needs to be cautious. Adult samples tend to consist of more chronically ill individuals and the differences in ASD findings could therefore indicate an illness effect, but equally could be maintaining factors of the eating disorder in those in whom ASD traits are observed. Illness effect could not be explored in the context of this review but could account for the heterogeneity of the included samples, or indeed whether longer illness duration elicits ASD traits. Therefore, further research is needed to determine whether the differences on the AQ and AQ-10 found between AN and HC samples extend to young people and if so, how this should be interpreted.

Analysis of the difference in sub-scale scores on the AQ between the AN and HC groups included only one child study (Calderoni et al. 2015) so it is not possible to draw conclusions about the difference between children and adults on sub-scale scores. The sub-scale analysis did suggest, however, that there was no significant difference in Calderoni et al.’s (2015) study between AN and HC groups on social skills, attention to detail, communication or imagination, again highlighting the need for further studies with both adult and child samples. Whilst analysis of the AQ sub-scales was limited by fewer studies and large heterogeneity, it indicated that that there was no significant difference between patients with AN and HCs on self-report attention to detail. This is contrary to consistent experimental, performance-based findings that individuals exhibit weaker central coherence, i.e. difficulties with bigger picture thinking relative to controls (For a review, see Lopez et al. 2008). However, Lopez et al. (2008) concluded that while those with AN have global processing difficulties, the findings on local processing were less clear. Thus, while people with AN may struggle with bigger picture thinking, they may not necessarily favour a detail focused approach. Of the seven items assessing attention to detail on the AQ, six referred to detail focused behaviour and only one referred to bigger picture thinking (Baron-Cohen et al. 2001). This may therefore indicate subtle differences between those with AN and individuals with elevated autistic traits or ASD in this domain.

Another key finding of this meta-analysis is that although AN patients were found to score significantly higher than HCs on the AQ and AQ-10, the mean scores were not high enough to meet the indicated cut-off for ASD, or as high as those with a diagnosis of ASD. For studies using the full version of the AQ, the mean scores ranged from 20.3 to 23.2, whereas 80 % of males and 92.3 % of females with ASD scored above 32 in the originally validation study (Baron-Cohen et al. 2001). Similarly, the studies using the AQ-10 reported mean scores of 3.89 to 4.05 which are below the cut-off of 6 (Allison et al. 2012). It therefore appears that the profile seen in AN represents an intermediate state between HCs and those with clinical ASD. This is not to say that scoring below 32 is not clinically significant. It is known that females with ASD may present differently to males. For example, Lai et al. (2011) found behavioural sex differences in ASD, as measured by the Autism Diagnostic Observation Schedule (ADOS), a standard clinical assessment tool for ASD, despite males and females not differing on childhood severity of core autistic symptoms. While the AQ was originally validated with just 13 females with Asperger’s/HFA (Baron-Cohen et al. 2001) and the scores of males and females in this group did not differ significantly, evidence suggests that the diagnostic profile of ASD may differ by gender, with females with HFA being less impaired in early social development (McLennan et al. 1993) and certain diagnostic items appear to be much more typical of girls than boys (Kopp and Gillberg 2011).

A more recent, large-scale study of 811 adults with ASD, 454 of whom were female (Baron-Cohen et al. 2014), found that AQ scores were higher in the ASD group than in HCs, with both sexes scoring above the suggested cut-off of 32. Males with ASD scored significantly higher than their female counterparts, indicating the presence of normative sex differences within ASD, adding strength to the notion that sex differences need to be considered in the clinical diagnosis of ASD. Lai et al. (2011) found that females with ASD had higher levels of self-report autistic traits as measured by the AQ, again suggesting a gender difference in the opposite direction of Baron-Cohen et al.’s (2014) large scale study.

One possibility is that gender differences in self-focused attention may influence how autistic traits are reported. For example, as females are more self-focused (Ingram et al. 1988) they may be more introspective than males, reported higher levels of autistic traits despite males having equal or more symptoms. This theory has not been tested empirically and is therefore an area which may warrant further research. Differences in self-focused attention may also not account for the differences in reported ASD traits between AN and HC groups as recent research suggests that individuals with AN have lower levels of self-focused attention than HCs, possibly accounted for by differences in executive function (Zucker et al. 2015). However, findings on gender differences in symptoms of ASD are not conclusive, and other research has found no gender difference in the symptoms of ASD, once IQ is controlled for (Holtmann et al. 2007). Further studies are therefore needed to address the differing presentation of ASD in males and females.

Regardless of whether the profiles of males and females with ASD are comparable, the presence of elevated levels of autistic traits in AN, as measured by the AQ or AQ-10, could still indicate the presence of a neurodevelopmental disorder prior to the onset of the eating disorder. Many of the domains measured by the AQ have been found to be impaired in both children and adults with AN including social skills and communication (Doris et al. 2014; Krug et al. 2013) and despite mixed findings, there is evidence of difficulties with attention switching and attention to detail in young people with AN (Lang et al. 2014a, b). Whilst these difficulties may not be severe enough to warrant exploration or diagnosis of ASD, they could still leave an individual vulnerable to the development of AN (Treasure and Schmidt 2013) and may impact on treatment outcome, making them clinically relevant (Nielsen et al. 2015).

Whilst five of the published studies used the full version of the AQ, only two used the brief AQ-10 so separate analysis of the studies using this version could not be performed. Although the effect size of the AQ-10 studies was still significant, it is not known whether either the AQ-10, or indeed the full version, are specific enough to differentiate between symptoms associated with ASD and those caused by co-morbidities such as obsessive compulsive disorder or social anxiety disorder. Fifty-six percent of inpatients with eating disorders have been found to also have an anxiety disorder (Blinder et al. 2006) and certain items on the AQ may tap into symptoms of anxiety rather than ASD per se. Similarly, self-report measures such as the AQ may not be sensitive enough to detect some of the subtle traits associated with female ASD, for example because of their ability to mask the social difficulties they may experience (Lai et al. 2011).

Relying on self-report measures to estimate the prevalence of ASD with AN may therefore lead to a false impression of the true incidence rate. A recent pilot study (Mandy and Tchanturia 2015) aimed to establish whether females with eating disorders who have social and flexibility problems meet ASD criteria. After assessing ten women using the ADOS 2nd Edition (ADOS-2; Lord et al. 2012), the standard assessment tool of ASD diagnosis, three received an ADOS-2 autism classification, a further two met criteria for ASD and two appeared to have ASD based on clinical reports but did not score above clinical cut-off on the ADOS. Thus, whilst there is evidence that even when using thorough, clinical assessment tools such as the ADOS-2 that a subset of women with AN also have elevated levels of ASD traits, it is not known whether the AQ or AQ-10 can accurately capture this. As the AQ and AQ-10 were designed as screening instruments, rather than diagnostic tools (Baron-Cohen et al. 2001), it may still be beneficial to use them in such a way within AN populations to identify individuals whom would benefit from a full ASD assessment. It would, however, benefit from further validity and reliability for use with this specific population. Future studies looking to address the issue of trait or state differences in levels of autistic traits in AN would benefit from using thorough clinical tools such as the ADOS along with developmental history in order to ascertain whether any difficulties associated with ASD were present prior to the onset of the eating disorder and whether they can be detected by clinical diagnostic tools.

The presence of elevated levels of autistic traits in AN may have important clinical and treatment implications. There is evidence that treatments such as Cognitive Remediation Therapy (CRT) effectively address some of the traits associated with ASD in AN including difficulties with set-shifting and central coherence (For a review see Tchanturia et al. 2014). More recently, Cognitive Remediation and Emotion Skills Training (CREST) has been developed to also target the socio-emotion difficulties found in ASD and AN (Tchanturia et al. 2015; Tchanturia et al. 2014). In the ASD field, work is also underway to tailor existing cognitive behavioural treatments to meet the needs of individuals with ASD and other psychiatric co-morbidities (Spain et al. 2015) and building on this work, there is scope to investigate the effectiveness of existing behavioural treatments for individuals with AN and elevated ASD traits. Additionally, the finding that a high proportion of females with AN also have high levels of autistic traits highlights the importance of considering gender differences in the diagnosis and treatment of ASD. ASD traits have been found to affect outcome in teenage-onset AN (Nielsen et al. 2015) and thus identifying these traits in an accurate and timely manner is of clinical importance.

## Conclusion

To our knowledge, this is the first review to attempt to synthesise existing literature on the use of the AQ and AQ-10 in AN populations. The results show that individuals with AN score significantly higher on the AQ than HCs but not high enough to reach the suggested clinical cut-off for ASD. Whilst this finding supports existing prevalence research and research into the similarities observed between people with AN and ASD, the results do not allow for conclusions to be drawn regarding whether a proportion of those with AN also have an underlying ASD. The self-report nature of the AQ and AQ-10, the presence of significant co-morbidities in AN and the effect of starvation on these symptoms make interpretation extremely difficult. Further research should use thorough, sensitive and validated clinical assessment tools to examine ASD in AN patients and the results of these should be compared to AQ or AQ-10 scores to explore the validity of using self-report measures to assess for ASD within this population.
